# Supplementary material for: Pyruvate Dehydrogenase Kinase 1 inhibition mediated oxidative phosphorylation enhancement in cartilage promotes osteoarthritis progression
Source: BMC Musculoskelet Disord. 2023 Jul 20;24:597. doi: 10.1186/s12891-023-06585-6 (PMC10357736; doi:10.1186/s12891-023-06585-6)
Supplement: Supplementary file 1 — Additional file 1. [file 12891_2023_6585_MOESM1_ESM.zip › MF.Gsea.1653627316242/GOMF_NUCLEOBASE_CONTAINING_COMPOUND_KINASE_ACTIVITY.html]

Details for gene set GOMF\_NUCLEOBASE\_CONTAINING\_COMPOUND\_KINASE\_ACTIVITY[GSEA]

|  || Dataset | 55457\_collapsed\_to\_symbols.cls.cls #OA\_versus\_normal.cls.cls #OA\_versus\_normal\_repos |
| Phenotype | cls.cls#OA\_versus\_normal\_repos |
| Upregulated in class | OA |
| GeneSet | GOMF\_NUCLEOBASE\_CONTAINING\_COMPOUND\_KINASE\_ACTIVITY |
| Enrichment Score (ES) | 0.55220705 |
| Normalized Enrichment Score (NES) | 2.0209246 |
| Nominal p-value | 0.0 |
| FDR q-value | 0.0070088725 |
| FWER p-Value | 0.077 |
Table: GSEA Results Summary

  

Fig 1: Enrichment plot: GOMF\_NUCLEOBASE\_CONTAINING\_COMPOUND\_KINASE\_ACTIVITY      
 Profile of the Running ES Score & Positions of GeneSet Members on the Rank Ordered List

  

| SYMBOL | TITLE | RANK IN GENE LIST | RANK METRIC SCORE | RUNNING ES | CORE ENRICHMENT || 1 | DGUOK | deoxyguanosine kinase [Source:HGNC Symbol;Acc:HGNC:2858] | 96 | 0.917 | 0.0722 | Yes |
| 2 | ADK | adenosine kinase [Source:HGNC Symbol;Acc:HGNC:257] | 127 | 0.878 | 0.1462 | Yes |
| 3 | NME7 | NME/NM23 family member 7 [Source:HGNC Symbol;Acc:HGNC:20461] | 178 | 0.835 | 0.2148 | Yes |
| 4 | NME5 | NME/NM23 family member 5 [Source:HGNC Symbol;Acc:HGNC:7853] | 362 | 0.720 | 0.2631 | Yes |
| 5 | AK1 | adenylate kinase 1 [Source:HGNC Symbol;Acc:HGNC:361] | 481 | 0.667 | 0.3118 | Yes |
| 6 | NME4 | NME/NM23 nucleoside diphosphate kinase 4 [Source:HGNC Symbol;Acc:HGNC:7852] | 677 | 0.599 | 0.3487 | Yes |
| 7 | CLP1 | cleavage factor polyribonucleotide kinase subunit 1 [Source:HGNC Symbol;Acc:HGNC:16999] | 748 | 0.582 | 0.3937 | Yes |
| 8 | NME3 | NME/NM23 nucleoside diphosphate kinase 3 [Source:HGNC Symbol;Acc:HGNC:7851] | 876 | 0.549 | 0.4316 | Yes |
| 9 | DCK | deoxycytidine kinase [Source:HGNC Symbol;Acc:HGNC:2704] | 920 | 0.537 | 0.4749 | Yes |
| 10 | NME2 | NME/NM23 nucleoside diphosphate kinase 2 [Source:HGNC Symbol;Acc:HGNC:7850] | 1428 | 0.439 | 0.4736 | Yes |
| 11 | GUK1 | guanylate kinase 1 [Source:HGNC Symbol;Acc:HGNC:4693] | 1640 | 0.407 | 0.4925 | Yes |
| 12 | PNKP | polynucleotide kinase 3'-phosphatase [Source:HGNC Symbol;Acc:HGNC:9154] | 1662 | 0.404 | 0.5260 | Yes |
| 13 | MPP1 | membrane palmitoylated protein 1 [Source:HGNC Symbol;Acc:HGNC:7219] | 1761 | 0.389 | 0.5522 | Yes |
| 14 | CASK | calcium/calmodulin dependent serine protein kinase [Source:HGNC Symbol;Acc:HGNC:1497] | 2316 | 0.324 | 0.5373 | No |
| 15 | DLG2 | discs large MAGUK scaffold protein 2 [Source:HGNC Symbol;Acc:HGNC:2901] | 2768 | 0.273 | 0.5260 | No |
| 16 | TK1 | thymidine kinase 1 [Source:HGNC Symbol;Acc:HGNC:11830] | 2909 | 0.259 | 0.5377 | No |
| 17 | DTYMK | deoxythymidylate kinase [Source:HGNC Symbol;Acc:HGNC:3061] | 3538 | 0.199 | 0.5062 | No |
| 18 | AK5 | adenylate kinase 5 [Source:HGNC Symbol;Acc:HGNC:365] | 3841 | 0.172 | 0.4976 | No |
| 19 | AK2 | adenylate kinase 2 [Source:HGNC Symbol;Acc:HGNC:362] | 4179 | 0.140 | 0.4836 | No |
| 20 | NME6 | NME/NM23 nucleoside diphosphate kinase 6 [Source:HGNC Symbol;Acc:HGNC:20567] | 4257 | 0.132 | 0.4891 | No |
| 21 | CMPK1 | cytidine/uridine monophosphate kinase 1 [Source:HGNC Symbol;Acc:HGNC:18170] | 5408 | 0.039 | 0.4031 | No |
| 22 | DLG1 | discs large MAGUK scaffold protein 1 [Source:HGNC Symbol;Acc:HGNC:2900] | 6219 | -0.022 | 0.3421 | No |
| 23 | NME1 | NME/NM23 nucleoside diphosphate kinase 1 [Source:HGNC Symbol;Acc:HGNC:7849] | 6328 | -0.031 | 0.3364 | No |
| 24 | AK6 | adenylate kinase 6 [Source:HGNC Symbol;Acc:HGNC:49151] | 6824 | -0.065 | 0.3036 | No |
| 25 | TK2 | thymidine kinase 2 [Source:HGNC Symbol;Acc:HGNC:11831] | 7468 | -0.116 | 0.2636 | No |
| 26 | AK4 | adenylate kinase 4 [Source:HGNC Symbol;Acc:HGNC:363] | 9682 | -0.290 | 0.1169 | No |
| 27 | TJP2 | tight junction protein 2 [Source:HGNC Symbol;Acc:HGNC:11828] | 9772 | -0.298 | 0.1359 | No |
| 28 | NOL9 | nucleolar protein 9 [Source:HGNC Symbol;Acc:HGNC:26265] | 10361 | -0.357 | 0.1212 | No |
| 29 | UCK2 | uridine-cytidine kinase 2 [Source:HGNC Symbol;Acc:HGNC:12562] | 10481 | -0.371 | 0.1442 | No |
| 30 | UCKL1 | uridine-cytidine kinase 1 like 1 [Source:HGNC Symbol;Acc:HGNC:15938] | 11515 | -0.503 | 0.1076 | No |
Table: GSEA details [plain text format]

  

Fig 2: GOMF\_NUCLEOBASE\_CONTAINING\_COMPOUND\_KINASE\_ACTIVITY      
 Blue-Pink O' Gram in the Space of the Analyzed GeneSet

  

Fig 3: GOMF\_NUCLEOBASE\_CONTAINING\_COMPOUND\_KINASE\_ACTIVITY: Random ES distribution      
 Gene set null distribution of ES for **GOMF\_NUCLEOBASE\_CONTAINING\_COMPOUND\_KINASE\_ACTIVITY**
